# Harmonization and integration of pharmacogenomics screens

**DOI:** 10.1093/bioinformatics/btag382

**Published:** 2026-06-12

**Authors:** Aleysha T Chen, Marcus R Kelly, Trey Ideker, Nicole M Mattson

**Affiliations:** Department of Bioengineering, University of California, San Diego, San Diego, CA 92093, United States; Department of Medicine, University of California, San Diego, San Diego, CA 92093, United States; Moores Cancer Center, University of California, San Diego, San Diego, CA 92093, United States; Department of Bioengineering, University of California, San Diego, San Diego, CA 92093, United States; Department of Medicine, University of California, San Diego, San Diego, CA 92093, United States; Moores Cancer Center, University of California, San Diego, San Diego, CA 92093, United States; Department of Computer Science and Engineering, University of California, San Diego, San Diego, CA 92093, United States; Department of Medicine, University of California, San Diego, San Diego, CA 92093, United States

## Abstract

**Motivation:**

Large pharmacogenomics screens have generated a wealth of information cataloguing the responses of more than a thousand tumor cell-line models to FDA-approved and exploratory drugs. Although centralized repositories have consolidated data access, the diversity of experimental platforms and response metrics used in these screens have made it challenging to integrate and compare their measured drug responses. Towards better pharmacogenomic data harmonization, we surveyed a range of data analysis protocols based on different curve-fitting functions (sigmoid, piecewise linear), different response metrics (IC50, EC50, integrated AUC), and different drug concentration windows (full range or truncated).

**Results:**

We found that an AUC derived from a sigmoidal curve fitted to a truncated dose range yields the strongest agreement between screening platforms, significantly bettering other protocols surveyed. This harmonization procedure also best aligns drug responses across successive iterations of the same platform. These findings broadly inform efforts to integrate drug response data in large-scale analyses.

**Availability:**

The source code to generate drug response profiles and correlations are available at https://github.com/digitaltumors/Pharmacogenomics_Screens_Harmonization.git.

## 1 Introduction

To date, thousands of small molecule compounds have been screened in more than a thousand genomically-sequenced cancer cell-line models to characterize dose response and resistance (Marcu and Marcu 2024, Nafchi *et al.* 2025). The accumulation of these drug response data holds immense potential for identifying biomarkers, elucidating drug mechanisms, and expanding cancer treatment strategies. Distinct pharmacogenomics screens have been generated by projects such as Genomics of Drug Sensitivity in Cancer (GDSC) (Garnett *et al.* 2012, Iorio *et al.* 2016), the Cancer Therapeutics Response Portal (CTRP) (Seashore-Ludlow *et al.* 2015), and Profiling Relative Inhibition Simultaneously in Mixtures (PRISM) (Yu *et al.* 2016, Corsello *et al.* 2020), among others (Alley *et al.* 1988, Barretina *et al.* 2012, Pemovska *et al.* 2013). To allow for easy access to available data, meta-repositories including PharmacoGx (Smirnov *et al.* 2016) and the Cancer Omics Drug Experiment Response (Coder) (PNNL-CompBio/coderdata: v2.1.0 n.d.) have consolidated all available data for end users. Access to the breadth of drug response data is a powerful asset in large-scale analyses for studying drug mechanisms and biomarker discovery.

In comparing these different repositories of data, the reported values of shared drug-cell line pairs are not always consistent (Haibe-Kains *et al.* 2013). Inconsistencies in drug response can arise from differences in cell growth conditions, concentrations of drugs tested, durations of drug treatments, or how cell viability was measured. To account for these variations, several groups have developed approaches to harmonize drug response profiles sourced from different datasets (Yadav *et al.* 2014, Cancer Cell Line Encyclopedia Consortium and Genomics of Drug Sensitivity in Cancer Consortium 2015, Bouhaddou *et al.* 2016, Mpindi *et al.* 2016, Pozdeyev *et al.* 2016, Safikhani *et al.* 2016). In addition, large pharmacogenomics datasets continue to emerge, prompting continued evaluation of best practices for data harmonization. For example, platforms such as PRISM (Yu *et al.* 2016, Corsello *et al.* 2020) not only make available a large drug screen but also offer the opportunity for individual laboratories to perform new screens using novel and/or proprietary drugs of interest (https://www.theprismlab.org/), an activity which greatly benefits from proper integration with prior drug screening data to place the new drugs in context.

**Figure 1:**
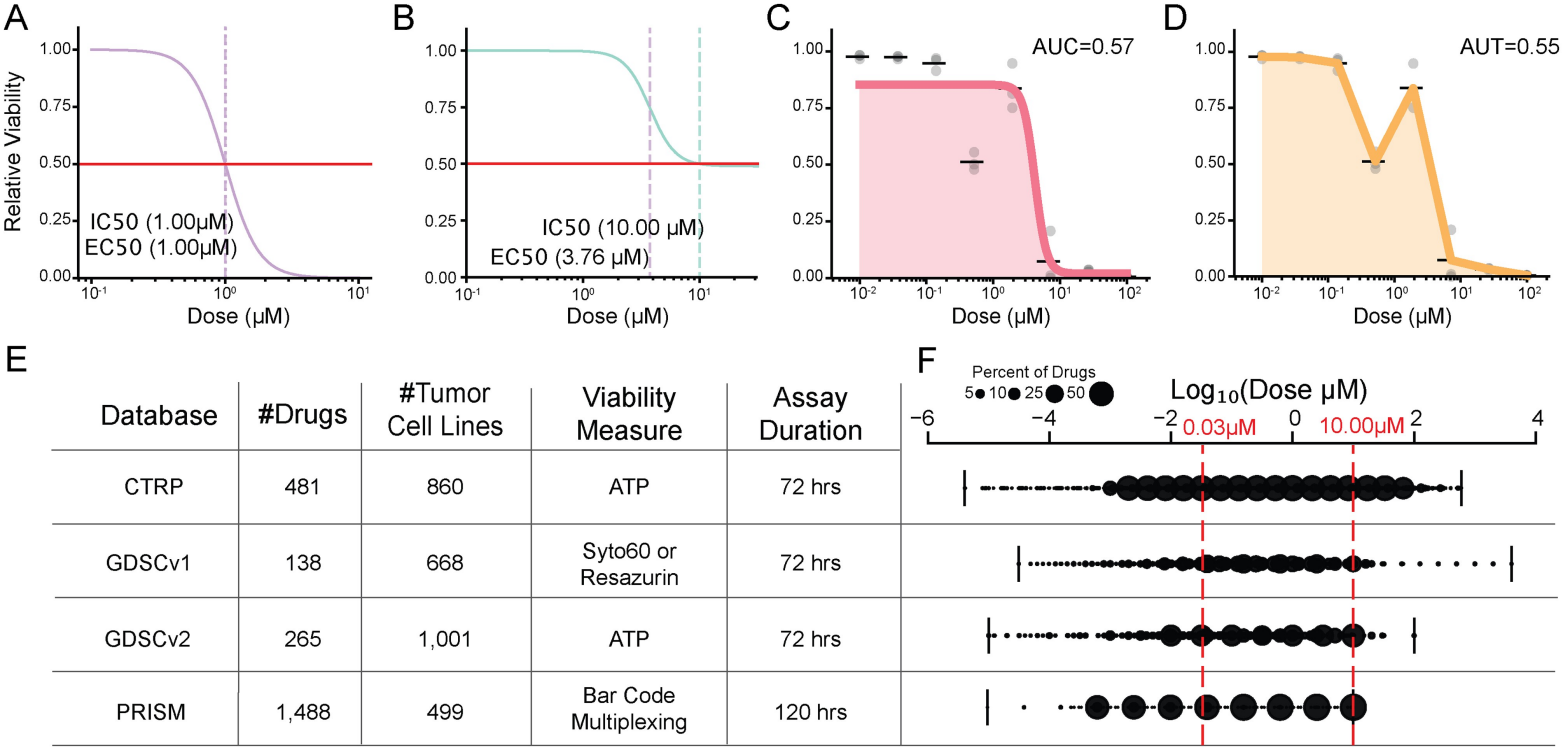
Overview of drug response scoring and databases. (A) Model sigmoidal drug response curve reaching full cell killing, for which IC50 and EC50 are identical. (B) Model sigmoidal curve reaching less than full cell killing, in which case the EC50 and IC50 can vary substantially. (C) Area under the dose response curve (AUC), computed by integrating a sigmoidal curve fitted to replicate viability values (grey dots) at progressive doses. Black horizontal bars represent the medians. (D) Alternative AUC calculated using the trapezoidal method (AUT), where an integral is taken under a piecewise linear function connecting the median viabilities at each dose (black bars). (E) Overview of the four databases compared in this study. Viability measures: ATP is measured by Cell Titer Glo™; Syto60 dyes nucleic acids (used for adherent cell lines); Resazurin is a compound reduced by viable cells (used for suspension cell lines); Bar Code Multiplexing is used to count the abundance of each cell line in pooled format. The assay duration is the length of drug treatment before measuring viability. (F) Dose ranges used by each database. Black vertical lines represent the dose minimum and maximum of each database. Dots show the specific drug doses used in a database, with dot size representing the percentage of drugs tested at a given dose. Red vertical dashed lines represent the truncated dose range used in this study.

Various metrics have been used to measure the response of a cell line to a given drug. The conventional approach involves fitting a sigmoidal curve to the relative cell viabilities observed at increasing drug doses. Different response metrics are then derived from this curve, for example the Inhibitory Concentration at 50% viability (IC50), the half maximal Effective Concentration (EC50), or the Area Under dose response Curve (AUC) (Meddings *et al.* 1989). The IC50 is the dose at which 50% cell viability is observed, whereas the EC50 is the dose corresponding to the midpoint in efficacy (Fig. 1A and B). The AUC is computed by integrating the sigmoidal curve between the minimum and maximum doses (AUC, Fig. 1C); instead of depending on a single concentration, this value reflects the cumulative drug effect across doses. However, a potential disadvantage of the aforementioned metrics is that calculations assume the data are well-modeled by a sigmoidal function–an assumption that can be readily violated by actual drug responses (Goutelle *et al.* 2008, Pozdeyev *et al.* 2016, Hafner *et al.* 2017). An alternative is to use a piecewise linear function, for instance as calculated by the “trapezoidal” method, which brings its own set of potential advantages and disadvantages (Campling *et al.* 1991) (AUT—Area Under Trapezoidal fit, Fig. 1D). Historically, AUC has been the main approach by which others [e.g., PharmacoGx (Smirnov *et al.* 2016)] have harmonized drug responses across the various pharmacogenomics databases.

In this study, we seek to evaluate the commonly used drug response measurements to identify the best approach to increase reproducibility among pharmacogenomics databases and in new releases of drug screening data. As we now describe, we find that AUC derived from a sigmoidal curve fitted to a truncated dose range (TruncAUC) markedly increases the reproducibility of drug response analysis over other approaches. Harmonizing the measured dose response curves within and between databases substantially widens the collections of available drugs and cell lines amenable to integrative analysis.

## 2 Methods

### 2.1 Pharmacogenomic data sourcing

Drug response datasets were downloaded from the following sources: CTRP (https://ctd2-data.nci.nih.gov/Public/Broad/CTRPv2.0_2015_ctd2_ExpandedDataset/), GDSCv1 and GDSCv2 (https://www.cancerrxgene.org/downloads/anova), and the PRISM Screen (https://depmap.org/portal/data_page/?tab=allData). Dose response data from PRISM runs 21–26 were obtained from the Broad Institute (sourced at https://doi.org/10.5281/zenodo.17196025). Drug names were harmonized across datasets via a list of known drug aliases, provided by each database, and standardized to one common name. Tumor cell-line names were harmonized by cross-referencing each database’s cell-line naming schemes and standardizing to those used by the Cancel Cell Line Encyclopedia (CCLE) (Barretina *et al.* 2012).

### 2.2 Computation of drug response profiles

We obtained drug response values $v_{p,d}(n,\;m)$ for each of nine profiles p ∈ {full_IC50, full_EC50, full_AUC, full_AUT, trunc_IC50, trunc_EC50, trunc_AUCsigmoid, trunc_AUT, AreaTrunc_AUC} and four databases d ∈ {CTRP, GDSCv1, GDSCv2, PRISM} over each of the available drug treatments n ∈ {up to 1,941 distinct drugs per database} and cell lines m ∈ {up to 1,300 distinct lines per database}. The pipeline used to calculate all drug response profiles (i.e., full- and truncated-range IC50, EC50, AUC, AUT, AreaTruncAUC) can be found at https://github.com/digitaltumors/Pharmacogenomics_Screens_Harmonization.git, <DrugResponseProfileFitCalculation.py>. A sigmoidal curve *y* was fit using relative viability as a log-logistic regression function of log_10_-transformed doses *x*, with the top asymptote fixed at 1:


(1) $$y=\mathrm{Bottom}+\;\frac{1-\mathrm{Bottom}}{1+{\left(\frac{10^{x}}{10^{\mathrm{logEC}50}}\right)}^{H}}$$

Bottom defines the bottom asymptote of the dose response curve, and H the Hill slope. If Bottom>0.5, the IC50 values are extrapolated by extending the sigmoidal curve and forcing convergence of Bottom=0 at high doses. All response profiles were normalized to common scales [log_10_(µM) for IC50 and EC50 and (0, 1) for integrated areas]. Calculated drug response profiles are reported in Table S1. IC50 and EC50 values are calculated from the log_10_-transformed doses and are reported in log_10_ space (Table S1). Truncation of the dose range did not change the fit quality of the sigmoidal curves (Fig. S5A and B available as supplementary data at *Bioinformatics* online; Table S1) from those fitted to a full dose range (Fig. S5C and D available as supplementary data at *Bioinformatics* online; Table S1).

**Figure 2:**
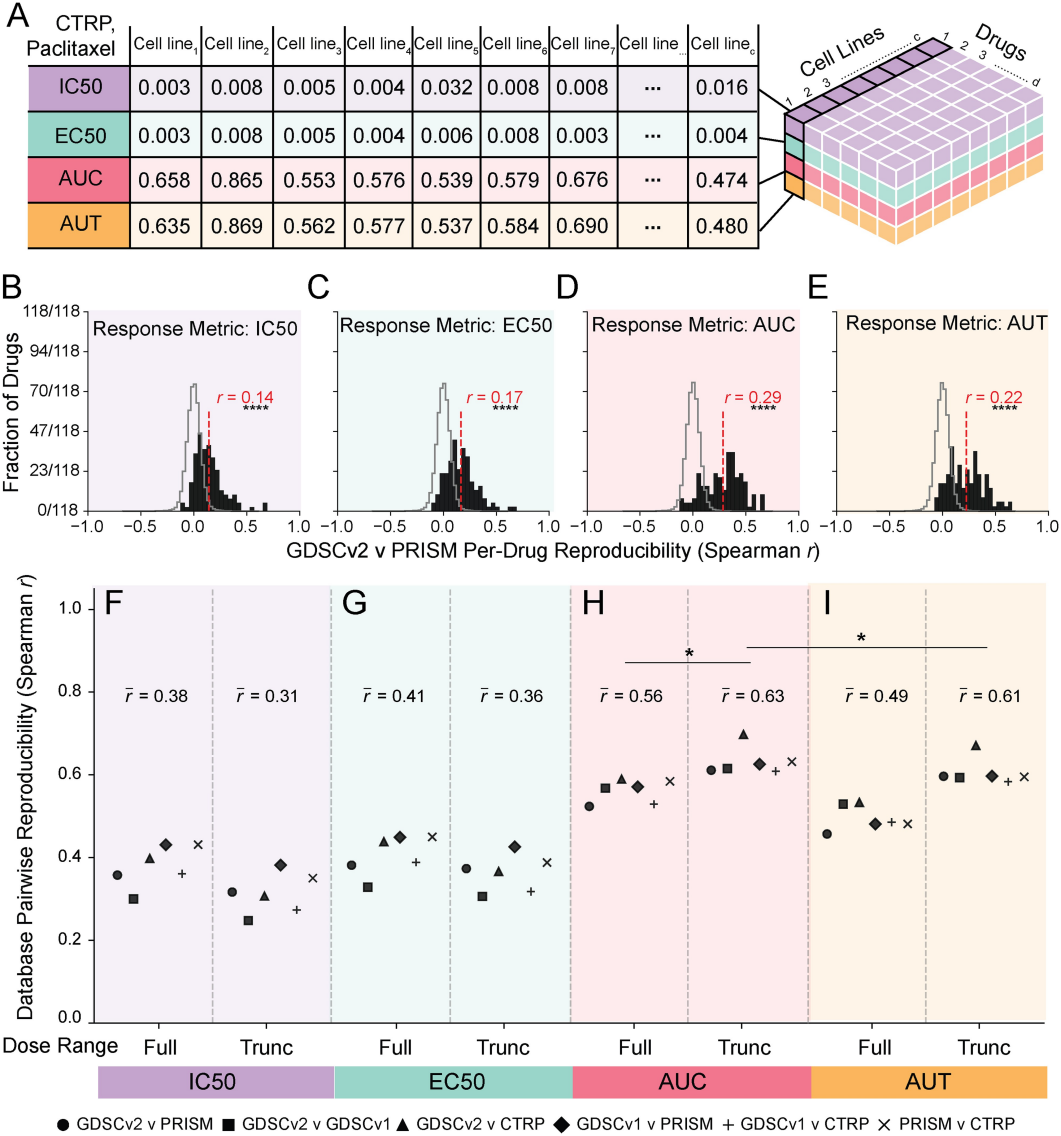
Assessment of reproducibility across pharmacogenomics databases. (A) Example drug response profile, using the drug paclitaxel from the CTRP database as an example. Rows: response profiles. Columns: cell lines. Far right: Each database represents a collection of response profiles for all drugs. (B) Per-drug reproducibility of IC50 response profiles between GDSCv2 and PRISM, tabulated over each of 118 drugs common to GDSCv2 and PRISM with valid values (filled histogram). Reproducibility measured by Spearman correlation. To create a negative control, IC50 values were permuted across cell lines and correlation recomputed over 10,000 permutations for each drug (hollow histogram). Mean values marked by a vertical dashed red line. **** *P* < 1 × 10^−4^ from a two-sided empirical permutation test. (C) As for panel B, but with EC50. (D) As for panel B, but with AUC. (E) As for panel B, but with AUT. (F) Database pairwise reproducibility of IC50 profiles as measured by the Spearman correlations (*r*) between pairs of databases (circle GDSCv2 v PRISM, square GDSCv2 v GDSCv1, triangle GDSCv2 v CTRP, diamond GDSCv1 v PRISM, plus-sign GDSCv1 v CTRP, x-sign PRISM v CTRP). Each correlation is computed across the matching drug response profiles for the two databases. In computing correlations, the full dose range is compared against a truncated dose range (x-axis, see text). Overall means of all database pairwise correlations are reported (*r¯*). * *P*  < 0.05 by a two-sided sign test. (G) As for panel F, but with EC50. (H) As for panel F, but with AUC. (I) As for panel F, but with AUT.

This same Python script was also used to compute drug response values for the successive PRISM runs 21–26. Given these data, the “response profile” of cell lines to a specific drug treatment (Fig. 2A) was defined as:


(2) $$v(n)=(v(n,\;m_{1})\;v(n,\;m_{2})\ldots \;v(n,\;m_{N}))$$

### 2.3 Reproducibility between databases

For a given drug-response profile and pair of databases d ∈ {x, y}, reproducibility of a drug treatment was defined by the Spearman or Pearson correlation r:


(3) $$r(v_{p,x}(n),\;v_{p,y}(n))$$

Such correlations were computed for each of all available treatments common to a given database pair, yielding a distribution of correlation values (filled histograms, Fig. 2B–E, Fig. S1 available as supplementary data at *Bioinformatics* online). We also computed empirical null distributions of reproducibility (hollow histograms, Fig. 2B–E, Fig. S1 available as supplementary data at *Bioinformatics* online). For this purpose, we performed 10,000 permutations k of response vector v for each of the common treatments, yielding permuted vectors $\pi_{k}(v_{p,x}(n))$. These permuted vectors were used to recompute all correlations in Equation 4. To calculate significance of the actual versus null correlations, we employed a two-sided empirical permutation test (Ernst 2004, Good 2004).

For each database pair we also computed the blanket reproducibility across all shared drug-cell line pairs (Fig. 2F–I):


(4) $$r(\mathrm{vec}(V_{p,x}),\;\mathrm{vec}(V_{p,y}))$$

where $V_{p,x}$ and $V_{p,y}$ are the N×M matrices of response values with elements $V_{p,x}[n,\;m]=v(n,\;m)$ and $V_{p,y}[n,\;m]=v(n,\;m)$ for drug n and cell line m common to both databases (Table S2). vec() denotes the vectorization operator that concatenates all drug-cell line pair response values. To attain 95% confidence intervals around each pairwise reproducibility, we bootstrapped Spearman correlations across 500 resamplings of the paired drug response profiles (Table S2). IC50 and EC50 reproducibility was calculated on the log_10_-transformed values (Table S2). Variability in IC50 and EC50 estimates for the same drug–cell line pairs can be attributable to differences in response magnitude and to whether IC50 or EC50 values fell within the experimentally tested dose ranges (Fig. S5E–H available as supplementary data at *Bioinformatics* online).

**Figure 3:**
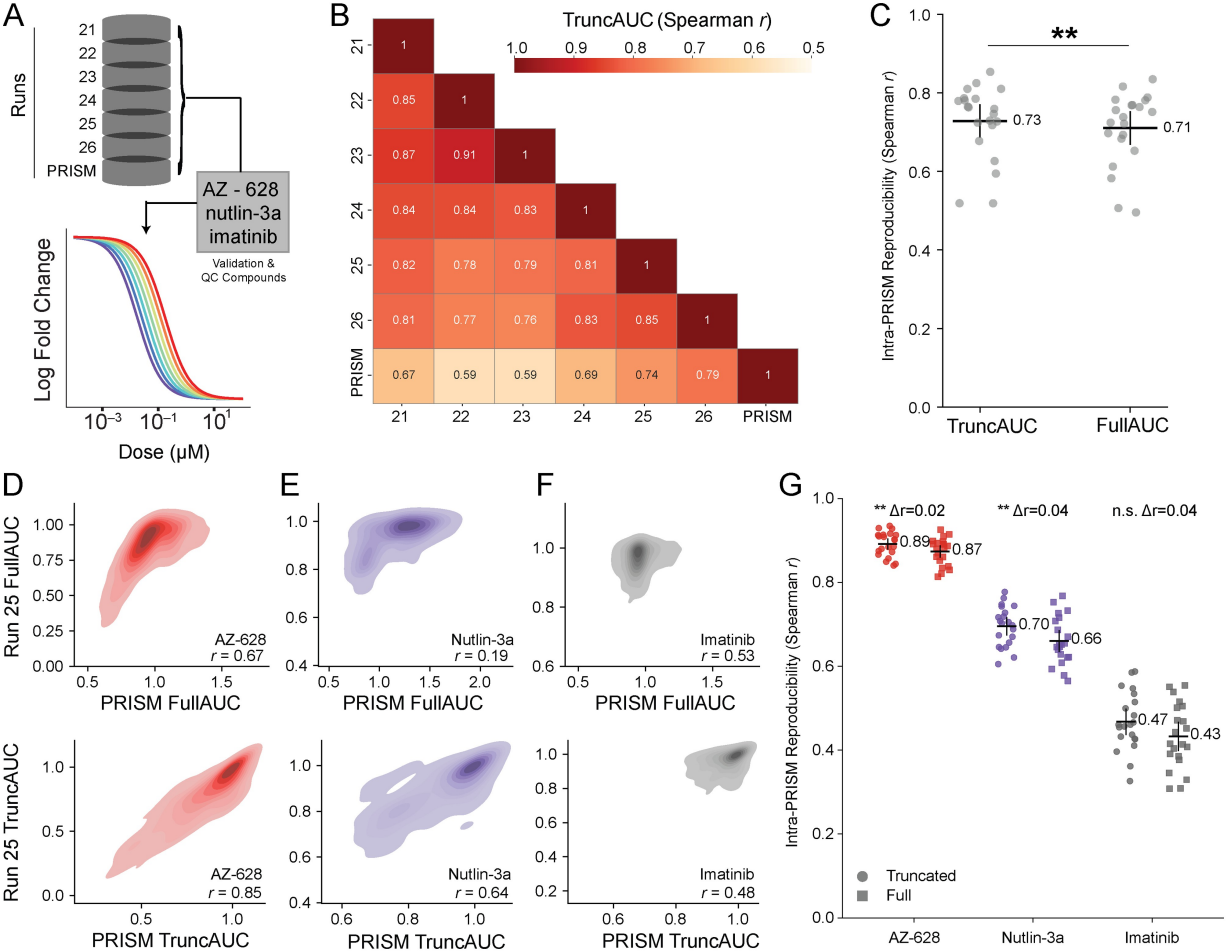
Alignment of iterative PRISM datasets. (A) Overview of seven successive runs on the PRISM platform [rows, the bottom row labeled “PRISM” represents the original published screen (Corsello *et al*. 2020)]. Cell lines are barcoded, pooled and quantified, reporting relative log fold changes in barcode representation. With each new drug submission, three “validation” drugs are included: AZ-628 (pan-RAF kinase inhibitor), nutlin-3a (MDM2 inhibitor), and imatinib (BCR-ABL and other tyrosine kinase inhibitor). (B) Drug response profiles are compared across pairs of PRISM runs (Spearman correlation of TruncAUC). Profiles for the three validation drugs are concatenated. (C) The pairwise reproducibility (grey dots) of the TruncAUC as compared to the FullAUC. Means denoted by black bars with 95% confidence intervals. ** *P* < 1 × 10^−3^ two-sided sign test. (D) Scatterplot (kernel density) of AZ-628 drug response for the original PRISM screen versus Run 25 (x vs. y). Top: FullAUC. Bottom: TruncAUC. (E) As for panel D, but with nutlin-3a. (F) As for panel D, but with imatinib. (G) Summary of the reproducibility results for each validation drug: AZ-628 (red), nutlin-3a (purple), and imatinib (grey) across all 21 pairs of PRISM runs (individual points) comparing TruncAUC (circles) to the original method of FullAUC (squares). Means denoted by black bars with 95% confidence intervals.** *P* < 1 × 10^−3^ two-sided sign test.

Reproducibility was also calculated for specific groups of drugs: effective drugs and drugs with 1 nM < IC50 < 100 µM. Effective drugs are subsetted as those with response values less than the median of the response profile. To compare the full and truncated drug response profiles we employed a two-sided sign (Dixon and Mood 1946) test (Figs. 2H, I and 3C, G; Fig. S6B, D, and E available as supplementary data at *Bioinformatics* online).

### 2.4 Identification of sensitive cell lines

Sensitive outlier cell lines were identified for each database by rank-ordering cell lines with increasing AUC and subsetting for the top 20 cell lines (Fig. S3A available as supplementary data at *Bioinformatics* online). For a given AUC response profile and pair of databases, the shared number of cell lines for a given drug was defined as j (Fig. S3B available as supplementary data at *Bioinformatics* online). To compare different drug response profiles, we employed a two-sided sign test (Dixon and Mood 1946) (Figs. S3C and S6C available as supplementary data at *Bioinformatics* online).

### 2.5 Alternative evaluations of reproducibility

Several alternative approaches were evaluated for assessing drug-response reproducibility. First, we implemented an alternative dose truncation method (AreaTruncAUC) which fits a sigmoidal curve to the full dose range but integrates only within the filtered dose range (0.03 µM to 10.00 µM) (Fig. S6A available as supplementary data at *Bioinformatics* online). AreaTruncAUC minorly improved cross-database reproducibility (Fig. S6B available as supplementary data at *Bioinformatics* online) but did not identify sensitive cell lines as effectively as TruncAUC (Fig. S6C available as supplementary data at *Bioinformatics* online). Second, we found that more effective drugs, defined as those with TruncAUC values below the median across all drugs, showed no significant improvement in reproducibility compared to the full drug set (Fig. S6D available as supplementary data at *Bioinformatics* online). Finally, we found that IC50 values restricted to a biologically relevant range (1 nM < IC50 < 100 µM) (Chen *et al.* 2025) increased reproducibility (Fig. S6E available as supplementary data at *Bioinformatics* online) but excluded 91,510 drug–cell line pairs, representing 42% of all available pharmacogenomics data (Table S3).

## 3 Results

### 3.1 Data sourcing

We sourced the major drug response datasets from CTRP, GDSC first and second releases (v1 and v2), and PRISM, encompassing 1,941 unique drugs and 1,300 unique tumor cell lines in total. We first surveyed the screening methodology used for each of these resources, observing distinct cell viability measures and assay durations (Fig. 1E). For example, CTRP and GDSC treated cells for three days whereas the treatment used in the PRISM screen lasted five days. Each resource also used specific assessments of cell viability: CTRP recorded levels of ATP within the live fraction of cells; GDSCv1 used either nucleus counting (Syto60, adherent cells) or metabolic competency (Resazurin, suspension cells); GDSCv2 measured ATP levels (similar to CTRP); and PRISM pooled and quantified populations of individually barcoded cells before versus after drug treatment. We also found that the drug dose ranges could vary substantially between databases, and different drugs could have differing series of doses within the same screen (Fig. 1F).

### 3.2 Cross-database reproducibility

We then examined how reproducibility across these databases is influenced by the calculation of drug response. For each drug, we applied four response metrics (IC50, EC50, AUC, AUT) across tumor cell lines to yield four alternative drug response “profiles” (Fig. 2A). This same procedure was carried out for each of the four databases, CTRP, GDSCv1, GDSCv2, and PRISM. We found that use of IC50 resulted in significant but relatively low observed per-drug reproducibility above the permuted null distribution, with a mean Spearman correlation of 0.14 per shared drug (Fig. 2B, Fig. S1 available as supplementary data at *Bioinformatics* online). Similarly for EC50, observed per-drug reproducibility was limited, with a mean Spearman of 0.17 per shared drug (Fig. 2C). In contrast, for both AUC (Fig. 2D) and AUT (Fig. 2E), we observed a greater per-drug reproducibility above the permuted null distribution with mean correlations of 0.29 and 0.22 per shared drug, respectively (Fig. 2D and E).

We next evaluated how database pairwise reproducibility (computed over all drug-cell line pairs) is affected by the range of drug concentrations used. Here two distinct protocols were evaluated: full versus truncated. The first protocol calculates each drug response using the aforementioned response metrics over the entire range of drug concentrations available (“full”). The second protocol truncates the range of drug concentrations to that used commonly by all databases prior to sigmoidal curve fitting and/or area integration (“trunc”). To define the truncated protocol, we found that a range of (0.03 to 10.00 µM) covered all drug screens performed by multiple databases preserving at least four doses per drug, while excluding a substantial number of atypically low or high measurements used by a particular database only (Fig. 1F). We found that dose truncation did not improve database pairwise reproducibility for IC50 and EC50 profiles (Fig. 2F and G), whereas it led to significant improvements for both AUC and AUT (Fig. 2H and I). Truncated AUC (TruncAUC) achieved the highest overall database pairwise reproducibility with an average *r *= 0.63 (Fig. 2H). Similarly, when Pearson correlation is used to measure reproducibility, IC50 and EC50 showed reduced database pairwise reproducibility (Fig. S2A and B available as supplementary data at *Bioinformatics* online) regardless of dose truncation. Pearson correlation-based reproducibility also reported improvements for both AUC and AUT with dose truncation, wherein TruncAUC reached greatest pairwise reproducibility (Fig. S2C and D available as supplementary data at *Bioinformatics* online).

Beyond correlation, a complementary means to assess reproducibility of a drug response profile is to examine the most sensitive cell lines identified for that drug in one database versus another (Fig. S3A available as supplementary data at *Bioinformatics* online). Here too, we observed that the TruncAUC metric resulted in superior cross-database agreement. For example, when identifying the top 20 cell lines sensitive to the drug AZD7762 in CTRP versus GDSCv2 databases, 10 sensitive lines were identified in common when using TruncAUC, versus 8 with FullAUC, the next best method (Fig. S3B available as supplementary data at *Bioinformatics* online). Extending this analysis across all drugs, we found that TruncAUC led to significantly greater reproducibility in its identification of drug-sensitive cell lines (Fig. S3C available as supplementary data at *Bioinformatics* online).

### 3.3 Reproducibility in successive iterations of the PRISM platform

Given the generally high reproducibility of TruncAUC, we sought to further evaluate this response metric in a complementary task–maximizing agreement among successive releases of drug screens from the PRISM platform. Since the initial PRISM dataset (Fig. 1E), this platform has been made available to the public for scoring drug responses of custom molecular compounds across 500+ tumor cell lines. In addition to new compounds, all PRISM screens include three standard drugs—AZ-628, nutlin-3a, and imatinib–which are used for validation and quality control across data releases (Fig. 3A). We obtained drug response data for these three compounds across a total of six additional PRISM screens (corresponding to PRISM runs 21—26, raw input data: https://doi.org/10.5281/zenodo.1,71,96,025 recalculated response values Table S4). We observed relatively high reproducibility across PRISM runs (Fig. 3B: TruncAUC), ranging in correlation from 0.59 (PRISM 23 versus original) to 0.91 (PRISM 22 to 23). Moreover, reproducibility by TruncAUC was significantly higher than that of other response metrics, including sigmoidal AUC with full dose range (FullAUC), the metric implemented by the original PRISM manuscript (Yu *et al.* 2016, Corsello *et al.* 2020) (Fig. 3C, Fig. S4 available as supplementary data at *Bioinformatics* online). For example, application of TruncAUC improved reproducibility of the AZ-628 drug response from a correlation of 0.67 to 0.85 (Fig. 3D). Drug response reproducibility was similarly improved for the other two validation drugs (Fig. 3E and F), and this pattern was consistent across PRISM runs (Fig. 3G).

## 4 Discussion

Here we have evaluated best practices for the harmonization of leading cancer pharmacogenomic datasets, results which are also relevant to integration of these resources with newly generated screening data. We systematically compared four different metrics of drug response: IC50, EC50, AUC, or AUT (Fig. 1A–D). For each database (Fig. 1E) every drug response was scored across every tumor cell line (Fig. 2A), yielding drug response profiles which were correlated with those from the other databases to assess reproducibility.

First, we observed that the area integration metrics, calculated either from a sigmoidal integration (AUC) or from a linear piecewise integration (AUT), consistently exhibit the greatest per-drug reproducibility between databases (Fig. 2B–E, Fig. S1 available as supplementary data at *Bioinformatics* online). Notably, database pairwise correlations (Fig. 2F–I) were systematically higher than per-drug correlations (Fig. 2B–E) because the former aggregates signals across many more drug-cell line observations, reducing drug-specific noise. Database pairwise reproducibility was maximal when the sigmoidal curve was fit using doses within the truncated dose range (TruncAUC) (Fig. 2H Spearman correlation, Fig. S2C available as supplementary data at *Bioinformatics* online Pearson correlation). As noted by earlier analyses, the choice of correlation coefficient can impact reproducibility (Cancer Cell Line Encyclopedia Consortium and Genomics of Drug Sensitivity in Cancer Consortium 2015). However, we show robustly that TruncAUC is the most reproducible metric for both coefficients. Furthermore, TruncAUC further provided significant improvement in identification of sensitive cell lines across databases (Fig. S3C available as supplementary data at *Bioinformatics* online).

In addition to the global harmonization of large pharmacogenomic databases, we also examined impacts of such harmonization on successive PRISM runs. When using TruncAUC, we observed relatively high reproducibility (*r* = 0.59 to 0.91, Fig. 3B) which was significantly higher than that obtained in the original PRISM screen using FullAUC (Yu *et al.* 2016, Corsello *et al.* 2020). The dose ranges of PRISM runs are not always identical, providing a clear rationale for the use of dose truncation in this case, and why it leads to improved reproducibility (Fig. 3C). Thus across multiple measures, TruncAUC provides the greatest reproducibility across datasets.

The weaker reproducibility of IC50 likely arises from differences in dose range coverage across databases, where the IC50 value falls in-range in one database (Fig. S5E and G available as supplementary data at *Bioinformatics* online) but is extrapolated in another (Fig. 5F and H). Such difficulties could also be attributed to variations in the viability assays used by each database (Fallahi-Sichani *et al.* 2013, Brooks *et al.* 2019, Niepel *et al.* 2019).

Our study is not the first to observe or attempt to address differences in screening outcomes among pharmacogenomics databases; others have proposed alternative methods that vary in complexity (Yadav *et al.* 2014, Geeleher *et al.* 2016, Hafner *et al.* 2016, Clark *et al.* 2017, Xia *et al.* 2022, Yingtaweesittikul *et al.* 2023). For example, Xia *et al.* implemented a variant of linear regression to bridge fixed-range AUC metrics across databases. Although increased drug response correlations were reported when using a constrained dose range, in a manner consistent with our own study, their linear regression model included additional parameters which do not further increase correlation beyond the simpler methods we have surveyed (Xia *et al.* 2022). Similarly, Pozdeyev *et al.* and Corsello *et al.* showed that drug response metrics become more consistent when using variable, shared dose ranges relative to the full range (Pozdeyev *et al.* 2016, Corsello *et al.* 2020). In contrast to these studies, we find good reproducibility using a unified range for all drugs without compromising curve fit quality (Fig S5A–D available as supplementary data at *Bioinformatics* online; Table S2), further simplifying the response profile calculation and its conceptual and computational overhead. Additionally, a unified dose range enables more direct cross-drug comparisons of tumor cell line responses.

In summary, we propose that calculating the AUC from a truncated dose range harmonizes drug response between pharmacogenomics databases as well as successive runs of the same screening platform. By improving such integration, this study increases the number of drugs and cell lines that can be cross-analyzed, further amplifying the utility of pharmacogenomics resources for drug and biomarker discovery.

## Supplementary Material

btag382_Supplementary_Data

## Data Availability

All full and truncated dose response profiles are available at <https://zenodo.org/records/20620409>, corresponding to Tables S1 to S4. Input data, including log_10_-transformed doses (µM) with relative variability of replicate level drug-cell line pairs for CTRP, GDSCv1, GDSCv2, PRISM, and PRISM runs 21–26, are available at <https://zenodo.org/records/17981848> and <https://zenodo.org/records/17196025>. Code related to generating the drug response profiles, correlation-based reproducibility, and null correlations can be found at <https://github.com/digitaltumors/Pharmacogenomics_Screens_Harmonization.git>.
