# Supplementary material for: Harmonization and integration of pharmacogenomics screens
Source: Bioinformatics. 2026 Jun 12;42(7):btag382. doi: 10.1093/bioinformatics/btag382 (PMC13330920; doi:10.1093/bioinformatics/btag382)
Supplement: btag382_Supplementary_Data [file btag382_supplementary_data.zip › Supplemental Figure Captions.docx]

**Supplemental Figure 1.** Observed versus permuted distributions of per-drug correlations for common drugs shared in pairwise comparisons of all four databases. (A) Per-drug reproducibility of IC50, EC50, AUC, and AUT response profiles tabulated over each of 113 drugs common to CTRP and GDSCv1, measured by Spearman correlation (filled histogram). To create a negative control, each drug’s profile values were permuted across cell lines, over 10,000 permutations (hollow histogram). Mean values marked by a dashed red line. **** P < 1 × 10^−4^ from a two-sided empirical permutation test. (B) As for panel A, but for 82 drugs common to CTRP v GDSCv2. (C) As for panel A, but for 126 drugs common to GDSCv1 v GDSCv2. (D) As for panel A, but for 197 drugs common to CTRP v PRISM. E) As for panel A, but for 162 drugs common to GDSCv1 v PRISM.

**Supplemental Figure 2**. Database reproducibility measured by Pearson correlation. (A) Database pairwise reproducibility of IC50 profiles as measured by the Pearson correlations (*r*) between pairs of databases (circle GDSCv2 v PRISM, square GDSCv2 v GDSCv1, triangle GDSCv2 v CTRP, diamond GDSCv1 v PRISM, plus-sign GDSCv1 v CTRP, x-sign PRISM v CTRP). Each correlation is computed across the matching drug response profiles for the two databases. In computing correlations, the full dose range is compared against a truncated dose range (x-axis, see text). Overall means of all database pairwise correlations are reported ($\bar{r}$). * P < 0.05 by a two-sided sign test. (B) As for panel A, but with EC50. (C) As for panel A, but with AUC. (D) As for panel A, but with AUT.

**Supplemental Figure 3.** Identification of sensitive cell lines by harmonization methods. (A) Rank plot of cell lines (points) ordered by TruncAUC-based sensitivity to AZD7762. Top 20 cell lines for each database are thresholded (vertical grey dashed lines) as sensitive outlier cell lines, out of which j cell lines are shared between databases (red). (B) Venn diagrams displaying j overlaps of the 20 most AZD7762-sensitive cell lines from CTRP or GDSCv2, ranked by truncated dose range AUC (TruncAUC) or full dose range AUC (FullAUC). (C) Kernel density plot of the number of sensitive cell lines for all drugs (n = 81) tested in both CTRP and GDSCv2. j sensitive cell lines identified with TruncAUC (y-axis) versus j sensitive cell lines identified with FullAUC (x-axis). p-value shown is derived from a two-sided sign test.

**Supplemental Figure 4.** Intra-PRISM reproducibility of different drug response profiles. (A). TruncAUC compared to the full dose range profiles of drug response calculated for IC50, EC50, and AUT. Each point represents a pair of PRISM runs. Black bars represent means with 95% confidence intervals, ** P < 1 × 10^-3^ two-sided sign test against TruncAUC. (B) As for panel A, with TruncAUC compared to the truncated dose range profiles of drug response calculated for IC50, EC50, and AUT. Black bars represent means with 95% confidence intervals, * P < 1 × 10^-2^ and *** P < 1 × 10^-4^ two-sided sign test against TruncAUC.

**Supplemental Figure 5.** Dose response curves with sigmoidal and trapezoidal fits and derived response metrics. Points represent relative viability observed at each dose. The fitted sigmoidal curve is represented in pink. The linear piecewise curve is represented in gold. (A) From GDSCv2, truncated dose range response of AGS cells treated with 781661-94-7 (sepantronium bromide). Grey dashed lines show lower (0.03µM) and upper (10.00µM) bounds of truncated dose range. (B) As for panel A, but from PRISM. (C) From GDSCv2, full dose range response of AGS cells treated with sepantronium bromide. (D) As for panel C, but from PRISM. (E) From GDSCv1, full dose range response of MCF7 cells treated with 781661-94-7 (sepantronium bromide). The IC50 and EC50 are shown on the curves as purple and green points, respectively. (F) As for panel E, but from GDSCv2. (G) From GDSCv2, full dose range response of YH13 cells treated with daporinad. (H) As for panel G, but from GDSCv1.

**Supplemental Figure 6.** Alternative evaluations of reproducibility. (A) A sigmoidal curve fitted to replicate viability values (grey dots) at progressive doses; black horizontal bars represent median viabilities. Area under the curve from integration (blue shaded region) within the truncated dose range. Grey dashed lines show lower (0.03µM) and upper (10.00µM) bounds. (B) Database pairwise reproducibility of AUC calculated from a curve fitted to the truncated dose range (TruncAUC) compared to truncating the area of full-range AUC (AreaTruncAUC). Each correlation is computed across the matching drug response profiles for the two databases. Black symbolized points each represent mean correlations of each database pair. Overall means of all database pairwise correlations are reported ($\bar{r}$). * P < 0.05 by a two-sided sign test. (C) Kernel density plot of the number of sensitive cell lines for all drugs (n=81) tested in both CTRP and GDSCv2. j sensitive cell lines identified with AreaTruncAUC (y-axis) versus j sensitive cell lines identified with TruncAUC (x-axis). p-value shown is derived from a two-sided sign test. (D) As for panel B, but comparing reproducibility of TruncAUC for all drugs versus effective drugs (TruncAUC < median TruncAUC of all drugs). n.s: not significant P = 0.22 by a two-sided sign test. (E) As for panel B, but comparing reproducibility of IC50 for all drugs versus drugs with 1nM < IC50 < 100µM. * P < 0.05 by a two-sided sign test.
